# Arch width changes in patients with Class II division 1 malocclusion treated with maxillary first premolar extraction and non-extraction method

**DOI:** 10.4317/jced.52840

**Published:** 2016-10-01

**Authors:** Sajjad Shirazi, Mojgan Kachoei, Naiemeh Shahvaghar-Asl, Samaneh Shirazi, Reza Sharghi

**Affiliations:** 1 Lecturer and Faculty Member, Faculty of Dentistry, Tabriz University of Medical Sciences, Tabriz, Iran; 2 Research Fellow, Dental and Periodontal Research Center, Tabriz University of Medical Sciences, Tabriz, Iran; 3 Associate Professor, Department of Orthodontics, Faculty of Dentistry, Tabriz University of Medical Sciences, Tabriz, Iran; 4 Post Graduate Student, Department of Pediatric Dentistry, Faculty of Dentistry, Tabriz University of Medical Science, Tabriz, Iran; 5 Under Graduate Student, Student Research Committee, Faculty of Paramedicine, Tabriz University of Medical Sciences, Tabriz, Iran; 6 Assistant Professor of Orthodontics, Dental Caries Prevention Research Center, Qazvin University of Medical Sciences, Qazvin, Iran

## Abstract

**Background:**

The aim of this study was to determine arch width changes during maxillary first premolars extraction and non-extraction treatment in patients with Class II division 1 malocclusion.

**Material and Methods:**

Dental casts of 91 Class II division 1 patients (36 males and 55 females) were evaluated. The minimum age of the subjects at the beginning of treatment was above 16 years. 48 patients were treated with extraction of the maxillary first premolars and 43 patients were treated without extraction. Pre- and post-treatment maxillary and mandibular inter-canine and inter-molar arch widths were measured.

**Results:**

At the end of treatment, maxillary and mandibular inter-canine widths of both groups increased significantly. The maxillary inter-molar width decreased in the extraction group and increased in the non-extraction group. The mandibular inter-molar width increased significantly in both groups. No significant differences were observed between males and females.

**Conclusions:**

The results of this study indicated that there was a tendency for an increase in arch width during both the extraction and non-extraction treatment except maxillary inter-molar width in the extraction cases.

** Key words:**Dental arch, malocclusion, angle Class II, tooth movement, extraction.

## Introduction

One of the major issues of concern and debate in orthodontics is the stability of achieved result (1). However, it is still a controversial issue maybe because it involves a multitude of intrinsic and extrinsic factors. The maintenance of inter-molar and inter-canine width has been widely discussed in the literature and is considered to be an important factor in getting stability after treatment (2).

Aesthetics is one of the main goals of orthodontic treatment. Narrower dental arches in extraction treatment, when compared with non-extraction, have been criticized in previous studies (3). However, the dental arch width at least in the canine area, is generally not smaller after extraction than after non-extraction treatment (3-5).

Bishara SE *et al.* (6) and Paquette DE *et al.* (4) evaluated the post-treatment results of extraction and non-extraction treatment in Class II division 1 patients and demonstrated a greater increase in inter-canine arch width within the maxillary and mandibular arches, in extraction group.

Burke SP *et al.* (1) in a meta-analysis noted that despite treatment modality or pre-treatment malocclusion, all patients experienced one to two millimetres expansion in mandibular inter-canine width during treatment.

The literature has provided evidence regarding the effect of extraction and non-extraction treatment. However, the findings on the extent of dental arch alterations in Class II extraction and non-extraction therapy display variation. This may be attributed to differences in amount of crowding, treatment modalities, amount of overjet, arch form, presence of displaced canines and the variability in sample sizes (7). Other effective factors that may affect treatment outcome are variations in the arch wire and treatment mechanics (7,8). Therefore, an attempt should be made to have homogenous study groups regarding these factors.

It has been recommended that the alteration in certain arch dimensions may be influenced by pre–treatment Angle classification and also treatment modalities (5,6,9-11). Accordingly, it is of crucial importance to investigate different classifications separately. In the literature, there are only few studies (4,6,10-13) which have evaluated arch width changes after orthodontic treatment in Class II division 1 subjects and all of these studies investigated four premolar extraction and non-extraction treatments.

This study was carried out to determine maxillary and mandibular arch width changes after orthodontic treatment in Angle Class II division 1 malocclusion subjects, treated either with or without extraction of the maxillary first premolars, and to compare the dental arch width changes in the treatment groups for both males and females. To the best of our knowledge the present study for the first time investigated the subjects treated with extraction in the maxillary arch and non-extraction in the mandibular arch.

## Material and Methods

-Subjects

This study was performed using pre and post-treatment study casts of consecutive subjects from the archives of the Department of Orthodontics between 2000 and 2014. The study design was in accordance with Helsinki Declaration on Ethical Principles for Medical Research Involving Human Subjects. The approval for the study was obtained from the IRB and research ethics committee of the university (Ref No. TBZMED.REC.1393.6).

Based on 80% power and significance level of 5% (14), and considering 1.5 as maximum tolerable error rate and based on standard deviation of 2.5, 45 samples were needed in each group. Patients who were treated either with or without bilateral maxillary first premolars extraction were included in this study considering the following criteria:

1. All cases were originally diagnosed as having mild to moderate skeletal Class II division 1 malocclusion.

2. None of the cases had congenital anomalies, significant facial asymmetries, or congenitally missing teeth.

3. All cases were above 16 years of age and all were in the permanent dentition.

4. All cases received no palatal expansion, functional appliance, orthognathic surgery or fixed prosthodontic therapy

5. All cases had overbite of 5% to 40% and mandibular arch crowding of ≤4 mm.

6. All cases were treated with fixed preadjusted (0.022-inch bracket slot) technique with class II elastics for non-extraction and space closure with sliding for extraction cases.

7. A clinically acceptable occlusion was established after active treatment i.e., a Class I canine relationship, an overbite between 10% and 25%, and well-aligned and inter-digitated arches.

8. Plaster dental casts were taken before and after orthodontic treatment.

These criteria were adopted to insure that post-treatment changes were not caused by poor treatment results.

-Study casts analysis 

**Figure 1:**
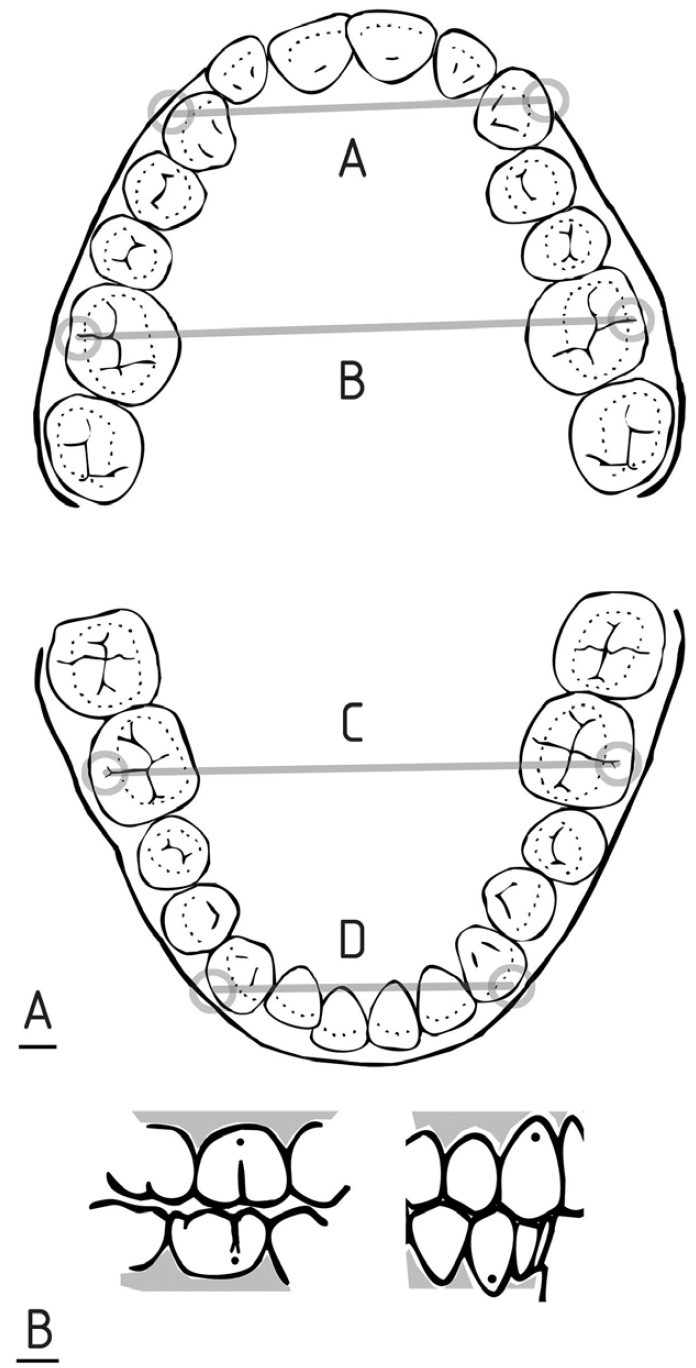
A) Maxillary and mandibular inter-arch measurements. B) Landmarks on the maxillary and mandibular dental cast.

Four arch width measurements were recorded from each subject’s dental casts using a digital calliper and recording the data to the nearest 0.1 mm. These measurements included: (A) maxillary inter-canine width between the height of contour points on the main buccal ridge located at the cervical third of the canines, (B) maxillary inter-molar width between the height of contour points located gingival to buccal grooves of the first molars, (C) mandibular inter-molar width between the height of contour points located gingival to main buccal pits of the first molars and (D) mandibular inter-canine width between the height of contour points on the buccal ridge located at the cervical third of the canines (Fig. 1A).

-Reliability of the measurements

Landmarks on the maxillary and mandibular dental casts were located and marked with a black 0.5 mm thick pencil. Each distance was measured by two examiners, on two occasions with a 2.5-month interval between the two measurements (Fig. 1B). Intra- and inter-examiner reliability was determined using intra-class correlation coefficients (ICCs).

-Statistical methods

Statistical analysis of the data was performed with SPSS for Windows version16 (IBM, Chicago, USA). A paired sample t-test was used to evaluate the treatment changes within each group. To compare the changes in the extraction and non-extraction groups for both males and females, independent student t-test was used. Statistical significance level was established at *P*<0.05.

## Results

A total of 91 Class II division 1 patients (36 males and 55 females; mean age=19.74±3.51) were included in the study. At the start of treatment, there was no statistically significant difference between arch widths of both groups (*P*>0.05). The sex distribution in groups was not significantly different (*P*>0.05). Almost perfect intra-examiner reliability for all examiners was determined (ICC = 0.96, 0.97); inter-examiner reliability was also perfect (ICC = 0.92).

-Evaluation of the upper arch

There were no statistically significant differences in maxillary dental arch width changes between males and females within the extraction group (*P*>0.05). This was the same for the non-extraction group ( Table 1).

**Table 1:**
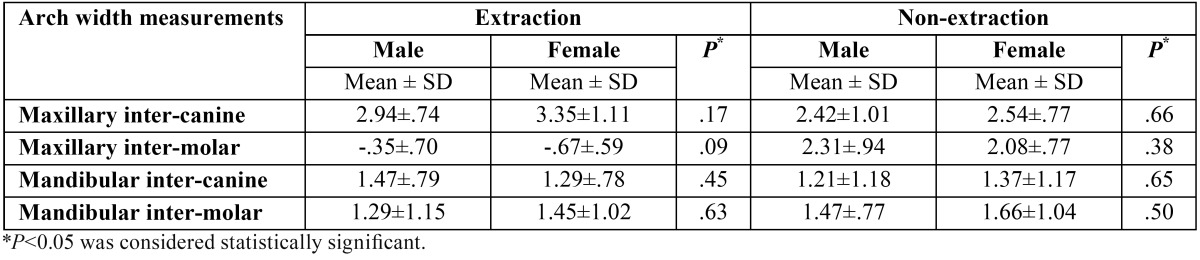
Comparison of dental arch width changes (mm) during treatment (T2-T1) between males and females treated with and without the extraction of maxillary first premolars.

At the end of treatment, the maxillary inter-canine widths increased significantly in both groups (*P*<0.001) ( Table 2). This increase was greater in the extraction group (3.2 ± 1 mm for the extraction group and 2.48 ± 0.88 mm for the non-extraction group) (*P*=0.001) ( Table 3). Comparing the extraction and non-extraction groups, females experienced a significantly greater increase in maxillary inter-canine width during treatment (T2-T1) in the extraction group ( Table 4).

**Table 2:**
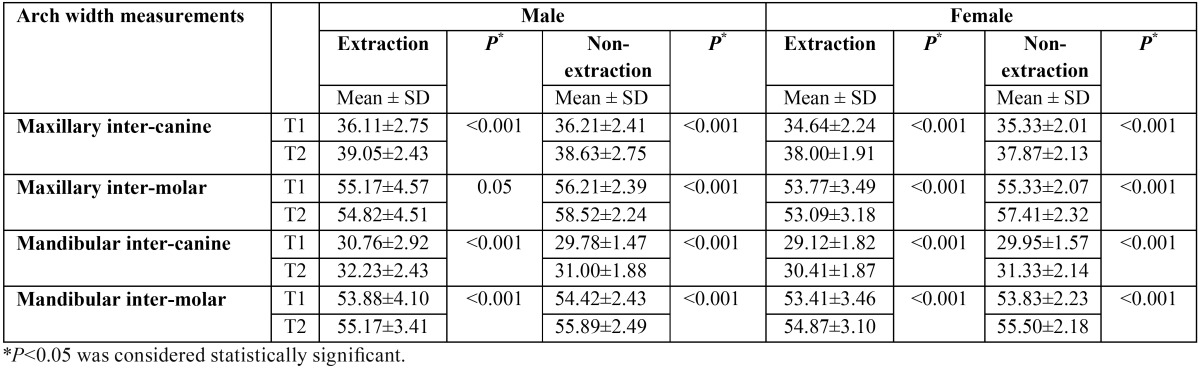
Comparison of pretreatment (T1) and post-treatment (T2) arch width averages (mm) in males and females treated with and without the extraction of maxillary first premolars.

**Table 3:**
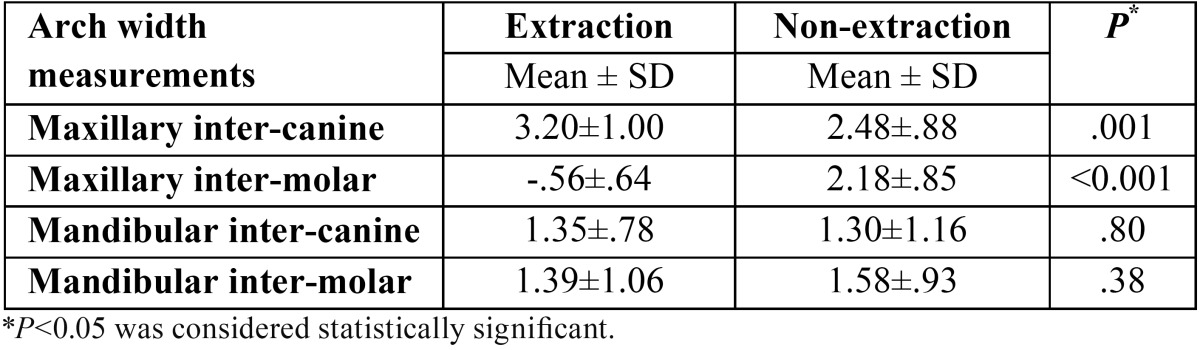
Comparison of dental arch width changes (mm) during treatment (T2-T1) between patients treated with and without the extraction of maxillary first premolars.

**Table 4:**
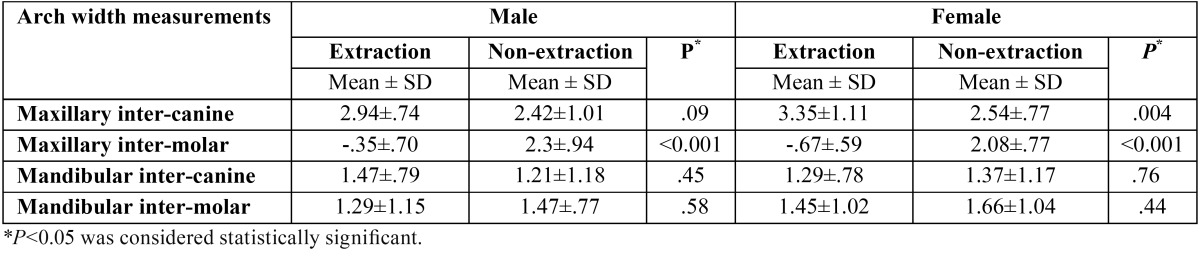
Comparison of dental arch width changes during treatment (T2-T1) for males and females treated with and without the extraction of maxillary first premolars.

Inter-molar width changes were in the opposite direction comparing the extraction and non-extraction groups (-0.56 ±0.64 mm for the extraction group and 2.18 ±0.85 mm for the non-extraction group) (*P*<0.001) ( Table 3). In the extraction group both males and females witnessed a decrease in inter-molar width during treatment. However, this decrease was not statistically significant in males (*P*>0.05). In the non-extraction group, inter-molar width significantly increased during the same period (*P*<0.001) ( Table 2). Similar trends were observed in males and females ( Table 4).

-Evaluation of the lower arch

No statistically significant differences regarding mandibular dental arch width changes were observed between males and females within both the extraction and non-extraction groups (*P*>0.05) ( Table 2).

Mandibular inter-canine and inter-molar widths significantly increased at the end of treatment (*P*<0.001) ( Table 2). The mean increase in inter-canine width was 1.35 ± 0.78 mm for the extraction group and 1.30 ± 1.16 mm for the non-extraction group (*P*>0.05). Inter-molar width increased 1.39 ± 1.06 mm in the extraction group and 1.58 ± 0.93 mm in the non-extraction group (*P*>0.05) ( Table 3). The trends were similar in males and females with no significant differences between extraction and non-extraction groups ( Table 4).

-Sex comparison 

The differences between males and females were not statistically significant (*P*>0.05). However, in general, dimensional increases or decreases tend to be greater in females.

## Discussion

Dimensional changes of the dental arches in both extraction and non-extraction treatments is well documented in the literature (3-8,11-13,15-17). However, these studies evaluated the treatment changes in four premolar extraction or non-extraction treatments.

An attempt was made in the present study to have a homogenous study group regarding malocclusion type, crowding and treatment mechanics. In this study we assessed and compared dental arch width changes in one type of malocclusion, Angle Class II division 1, thus maintaining this variable constant.

The subjects were treated either with or without bilateral maxillary first premolars extraction. To the best of our knowledge this is the first study concerning this method of treatment. In this study, arch width changes in treatment groups were also investigated for males and females separately. However, it should be noted that this study only relates to the treatment findings and that the long-term stability of the treatment approaches may lead to differences between the groups in terms of relapse and post-treatment changes (18).

The anatomic height of contours of the canines and molars were chosen as the measuring landmarks instead of the more customary cusp tips or the most buccal points for two reasons: 1- to eliminate dimensional alterations caused by the buccolingual inclinations of the related teeth (18), 2- to prevent confusion when selected cusps tips were not distinct (3).

The assessment of the data for the upper inter-canine distance showed a significant increase in all treatment groups regardless of the treatment modalities. It was also found that the inter-canine distance increased more in the extraction group than in the non-extraction group which is in agreement with earlier findings (4-6). This greater increase may be a consequence of the buccal and distal movement of the canines on the alveolus into the extraction site, there by occupying a broader part of the arch (1).

The difference among the groups regarding maxillary inter-molar arch width change arises from different treatment methods. In the non-extraction group, there was a statistically significant increase in the maxillary inter-molar width, whereas the extraction of two maxillary premolars caused a statistically significant decrease in this measurement, reflecting the mesial movement of these teeth to the narrower anterior part of the arch in extraction treatment (10,18). These results support earlier work by Luppanaporn-larp S *et al.* (5) who reported a decrease in maxillary inter-molar width in extraction Class II patients. In another study by Boley JC *et al.* (19), the inter-arch changes of four premolar extraction in Class I cases were assessed. According to their findings, maxillary inter-molar widths decreased significantly. However, the differences in treatment approaches and malocclusion types in these studies should be considered.

In the lower arch, inter-canine and inter-molar arch widths increased significantly in both treatment groups. These are in line with results of previous studies (4,6,10,12,13). Since the treatment method does not include any extraction in the lower arch, these outcomes can be explained by minimal expansion with the arch wires (7).

In the extraction group the mandibular inter-canine width showed greater increase. It may be a result of the greater increase of maxillary inter-canine width in the extraction group. Even though, it was not statistically significant when compared with the non-extraction group. This finding was also reported by other studies (4-6). However, the extraction method was different in these studies.

Taner TU *et al.* (12) in an evaluation of arch width changes after non-extraction treatment observed an increase in Class II division 1 cases in maxillary and mandibular inter-canine and inter-molar widths. Luppanapornlarp S *et al.* (5) reported that maxillary and mandibular inter-canine width increased during both extraction and non-extraction treatments in Class II patients. They also found minor expansion in the mandible compared with the maxilla in non-extraction treated patients. It might be due to pre-treatment maxillary arch form of the patients with Class II Division 1, which is in general tapered requiring more expansion. These compare very favourably with our results.

Kim E *et al.* (10) studied the arch width changes of extraction and non-extraction groups and found that extraction treatment does not result in narrower dental arches than non-extraction treatment. Isik F *et al.* (18) in a similar study reported the same results as Kim E *et al.* (10) with the exception of a decrease in lower inter-canine width in the non-extraction group. Similarly, the findings of the present study are in line with those of Kim and Gianelly. However, the extraction and non-extraction groups in these two earlier studies were not homogenous. Thus, the results reported in these studies might have been influenced by the wide range of individual variations (7).

Bishara SE *et al.* (11) compared the dental arch changes in patients with Class II division 1 malocclusions between the extraction and non-extraction groups and found that there was a tendency for an increase in inter-canine widths in both groups during treatment . The maxillary and mandibular inter-molar widths increased in the non-extraction group and decreased in the extraction group. In a similar study by Paquette DE *et al.* (4), same trends were reported except that maxillary inter-molar width was maintained in the extraction group. In the present study, however, the mandibular inter-molar width in the extraction group increased which can be attributed to the differences in the type of extraction used in this study.

In a study of the nature of normal changes in maxillary and mandibular arch width of an untreated population, from the early mixed dentition to the permanent dentition (from 7 to 15 years of age), an increase of about 2.5 mm in transpalatal width between the upper first molars has been reported (20). Other Investigators who studied growth changes in the transverse arch width found that molar and canine arch widths did not change after age 13 in female subjects and age 16 in male subjects (21-23). Mean ages at the beginning of treatment in previous discussed studies were between 11 and 14.3 years (4-7,10,12,13,18). Therefore, in these studies it was not possible to differentiate the treatment effects from natural growth especially in male subjects, because arch widths changes were evaluated in patients below the appropriate age range. The minimum age of the subjects chosen for the present study, at the start of treatment, was 16 years old. Therefore, the effect of growth and development on transverse arch width was not of concern. This might be one of the most distinguishing features of the present study.

The results of this study indicated that there is a tendency for an increase in arch width during both the extraction and non-extraction treatment in Class II division 1 malocclusions except maxillary inter-molar width in the extraction cases. In general, when comparing the findings of this study with those of other studies, it seems that the direction of treatment changes is similar in arches treated with extraction in the Class II cases. This comparison is also true for arches treated with non-extraction method. Nevertheless, it should be borne in mind that when treating a patient with extraction in one arch and non-extraction in the other arch, the two arches can influence each other. One might argue that there might be selection bias at the start of the study because of its retrospective design. However, it should be mentioned that the records of all consecutive patients, from 2000 to 2014, fulfilling the inclusion criteria were evaluated in order to avoid selection bias. Further prospective studies controlling for probable confounding factors are warranted.

In the extraction cases, if we assume that the arch dimensional changes are because of tooth movement into narrower or wider parts of the arch, then different anchorage values and different arch forms will cause different changes in arch width between identical teeth. This needs to be investigated in future studies.
